# Echocardiographic AV-interval optimization in patients with reduced left ventricular function

**DOI:** 10.1186/1476-7120-2-30

**Published:** 2004-12-17

**Authors:** C Melzer, AC Borges, F Knebel, WS Richter, W Combs, G Baumann, H Theres

**Affiliations:** 1 I Medizinische Klinik mit Schwerpunkt Kardiologie, Angiologie und Pulmologie, Charité, Campus Mitte, Berlin Germany; 2 Klinik für Nuklearmedizin, Charité, Campus Mitte, Berlin Germany; 3 Medtronic Inc., Minneapolis, USA

## Abstract

**Background:**

Ritter's method is a tool used to optimize AV delay in DDD pacemaker patients with normal left ventricular function only. The goal of our study was to evaluate Ritter's method in AV delay-interval optimization in patients with reduced left ventricular function.

**Methods:**

Patients with implanted DDD pacemakers and AVB III° were assigned to one of two groups according to ejection fraction (EF): Group 1 (EF > 35%) and Group 2 (EF < 35%). AV delay optimization was performed by means of radionuclide ventriculography (RNV) and application of Ritter's method.

**Results:**

For each of the patients examined, we succeeded in defining an optimal AV interval by means of both RNV and Ritter's method. The optimal AV delay determined by RNV correlated well with the delay found by Ritter's method, especially among those patients with reduced EF. The intra-class correlation coefficient was 0.8965 in Group 1 and 0.9228 in Group 2. The optimal AV interval in Group 1 was 190 ± 28.5 ms, and 180 ± 35 ms in Group 2.

**Conclusion:**

Ritter's method is also effective for optimization of AV intervals among patients with reduced left ventricular function (EF < 35%). The results obtained by RNV correlate well with those from Ritter's method. Individual programming of the AV interval is fundamentally essential in all cases.

## Background

Since introduction of the DDD pacemaker in the early 1980s, researchers have repeatedly attempted to optimize the atrioventricular (AV) interval, for the purpose of maximizing patient hemodynamic performance. Cannon waves may be induced by programming excessively short AV intervals, and diastolic mitral regurgitation may occur with excessively long programmed AV intervals. The AV interval is considered optimal (AV_opt_) if it allows maximum cardiac output.

The duration of the optimal AV interval varies throughout a wide range among individuals, primarily the result of appreciable differences in interatrial conduction [1-4].

An extensive variety of techniques has been employed to optimize AV delay, including acquisition and analysis of essential hemodynamic parameters by means of aortic-valve Doppler signals, impedance cardiography [9-11], Swan-Ganz catheterization [12-15], and especially the stroke volume [5-8]. Leman et al. [16] have demonstrated that it is also possible to utilize measurement of left ventricular ejection fraction and stroke volume by myocardial thallium scintigraphy as a means of AV interval optimization. A further possibility involves detection of left atrial depolarization by an esophageal electrode recording [17,18]. During recent years, the use of Doppler echocardiography in conjunction with the mitral valve inflow profile has been investigated as means of AV interval optimization: i.e., Ritter's method [19]. Previous investigations have evaluated Ritter's method in patients with normal left ventricular ejection fractions. During recent years, cardiac resynchronization therapy (CRT) has increasingly gained in significance for patients with chronic heart failure (CHF) [20]. In cases without ventricular desynchronicity, normal DDD pacemakers (or ICDs with DDD pacemaker function) will in future continue to be implanted in patients with reduced left ventricular ejection fraction. The goal of our study was accordingly to apply Ritter's method – until now validated only for patients with normal EF – for patients with reduced left ventricular ejection fraction (EF < 35%).

## Methods

We studied 20 DDD pacemaker patients within the context of in-office follow-up. Table 1 shows the baseline characteristics and Table 2, the inclusion criteria. We classified patients into two groups, according to left ventricular ejection fraction results obtained by echocardiography. Group 1 consisted of 10 patients with normal left ventricular ejection fraction, or with moderately reduced EF (EF > 35%). Group 2 comprised 10 patients with appreciably reduced left ventricular ejection fraction (EF < 35%).

**Table 1:** Clinical characteristics of the patients

**Characteristics**	**Group 1 (EF > 35%) **(*n *= 10)	**Group 2 (EF < 35%) **(*n *= 10)
Age	68.5 ± 4.5	65.7 ± 6.3
Male sex (%)	60	100
Left ventricular ejection fraction (%)	58 ± 9.7	22 ± 7.4
Left ventricular end-diastolic dimension (mm)	47	61
Coronary artery disease (%)	30	50
Dilated cardiomyopathy (%)	0	50
Hypertension (%)	30	0
Pharmacologic therapy (%)		
ACE inhibitor	40	100
Beta-blocker	50	90
Loop diuretic	0	100
Spironolactone	0	70

**Table 2:** Inclusion criteria

DDD pacemaker by AVB III° with permanent atrial and ventricular pacing
No left bundle-branch block or possible indication for CRT
Pacemaker implantation at least 4 months beforehand
NYHA Class I or II

We performed ejection fraction analysis by RNV and Ritter's method to achieve AV optimization, for 5 AV intervals in the range of 100 to 250 ms. We performed all measurements within 15 minutes of AV interval programming for every patient. All patients were permanently stimulated in the right atrium and right ventricle (binodal disease). Patient heart rate remained constant during the measurement period at programmed pacemaker lower rate (60 – 70 beats/min).

### Analysis of left ventricular ejection fraction by RNV

We performed radionuclide ventriculography (RNV) after in vivo marking of erythrocytes with tin DTPA and 10 MBq/kg KG Tc-99m, using a single-head Gamma camera (CGR gammatome 2, General Electrics, Paris, France) with a high-resolution, medium-energy collimator. For RNV we applied the equilibrium technique at 16 frames per cycle with patients at rest, and ventricular pacing at programmed AV intervals. We acquired a minimum of one million counts per image, and stored the data in a 64 × 64 matrix.

We calculated left ventricular ejection fraction (LVEF) semi-automatically after spatial and temporal smoothing and background subtraction. After Fourier analysis of ventricular stimulation progression, we recorded (with examiner definition) a region of interest (ROI) around the end-diastolic contour of the left ventricle and calculated the LVEF as follows:



### Ritter's method

By 1994 a method developed by Ritter et al. had become established for optimizing the AV interval [19]. A prerequisite for application of Ritter's method is Doppler-echocardiographic measurement of the mitral inflow profile.

Ritter's method employs the following formula for calculation of the optimal AV interval:

*AV*_*opt *_= *AV*_*long *_- (*a *- *b*)

**Figure 1:**
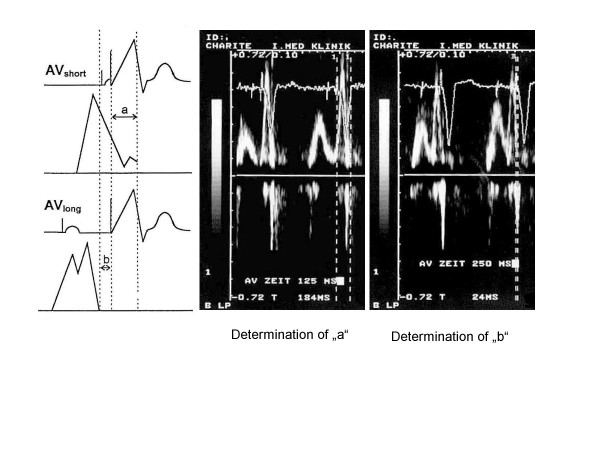
Ritter's method: The first step is determination of "a" for a nonphysiologically short AV interval (e.g. 125 ms), followed be determination of "b" for a nonphysiologically long AV interval (e.g. 250 ms).

We applied the following procedure in application of this formula (see Fig. 1):

#### Step 1

The first step involves programming for the pacemaker a nonphysiologically short AV interval, followed by determination of "a". This value "a" is the temporal interval between the ventricular contraction spike and the end of the A wave. "a" designates the electromechanical delay between right ventricular stimulation and the beginning of the left ventricular systole (i.e., closure of the mitral valve).

#### Step 2

The next step is programming for the pacemaker a long AV interval (*AV*_*long*_), followed by determination of "b". This value "b" is the temporal interval between the ventricular contraction spike and the end of the A wave. *AV*_*long *_- b defines the duration of the undisturbed maximal diastolic left ventricular filling.

The purpose of AV interval optimization in accordance with Ritter is to allow the ventricular systole to begin immediately subsequent to maximum, undisturbed diastolic ventricular filling and, in turn to prevent the occurrence of Cannon waves as well as diastolic mitral regurgitation.

### Statistics

We applied intra-class correlation in performing statistical evaluation.

## Results

### Group 1

**Figure 2:**
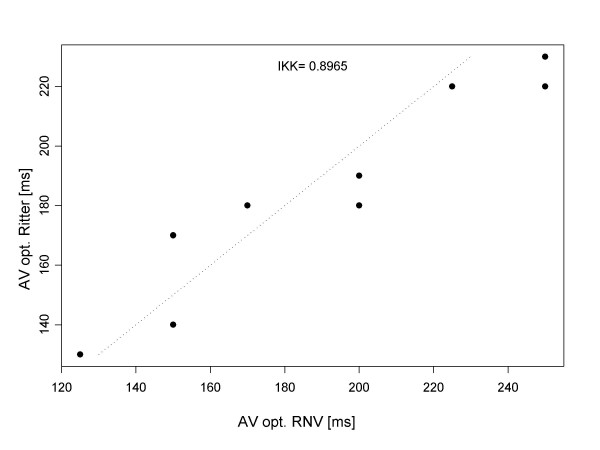
The correlation of the results of the RNV and Ritter methods, with respect to the optimal AV interval for Group 1.

**Figure 4:**
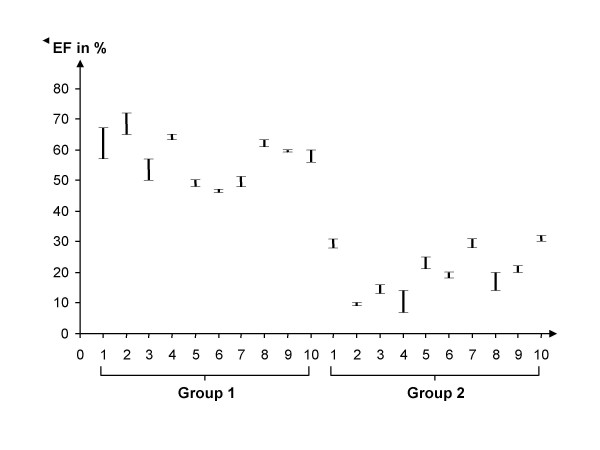
The maximum difference in left ventricular EF, determined by RNV and as a function of the programmed AV interval, for each of the patients examined.

In a given patient, our results indicated that it was possible to define an optimal AV interval for every patient: both by RNV as well as by Ritter's method. The mean optimal AV interval was 190 ± 28.5 ms. The correlation between RNV and Ritter's method is good: the intra-class quotient is 0.8965 (see Fig. 2). In results calculated by RNV, the mean percent difference in left ventricular ejection fraction between the hemodynamically best and worst AV intervals was 11 ± 4% (see Fig. 4).

### Group 2

**Figure 3:**
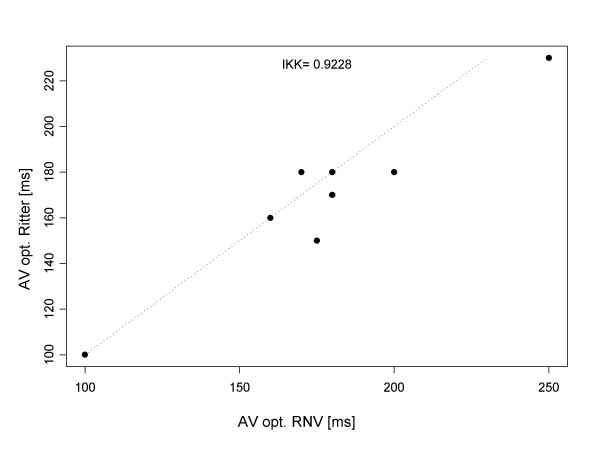
The correlation of the results of the RNV and Ritter methods, with respect to the optimal AV interval for Group 2.

In a given patient, we likewise succeeded in defining the optimal AV interval for every patient: both by RNV as well as by Ritter's method. The mean optimal AV interval was 180 ± 35 ms. In Group 2 as well, there was good correlation between RNV and Ritter's method: the intra-class quotient was 0.9228 (see Fig. 3). In results calculated by RNV, the mean percent difference in left ventricular ejection fraction between the hemodynamically best and worst AV intervals was 28 ± 11% (see Fig. 4).

## Discussion

A number of studies have documented the importance of AV synchronization for maximizing the left ventricular ejection fraction in pacemaker patients [21-24]. Despite CRT, the implantation of a DDD pacemaker (or ICD with DDD-pacemaker function) is still justified for patients with reduced left ventricular function and a lack of ventricular asynchrony.

The goal of our study was accordingly to apply Ritter's method for patients with reduced left ventricular ejection fraction. In every subject of Groups 1 and 2, it was possible on the basis of the left ventricular ejection fraction to define the optimal AV delay by means of RNV. The method of AV delay optimization by RNV has been previously verified [16]. The cost and complexity of this method, however, have hindered its extensive clinical application. On the basis of minimal inter- and intraobserver variability this method is nevertheless very well suited as a reference method.

Our application of Ritter's method enabled definition of the optimal AV interval for all patients. We further determined that Ritter's method can be reliably employed even in cases of reduced left ventricular systolic function. The AV interval calculated by Ritter's method correlated well with data obtained by RNV: both for normal (with intra-class coefficient of 0.8965) as well as for reduced left ventricular EF (intra-class coefficient of 0.9228).

Since Ritter's initial publication in 1994, AV interval optimization on the basis of the mitral valve inflow profile has been reported in one additional study [19]. In 1997 Kindermann et al. compared results calculated from Ritter's formula with those obtained from impedance cardiography [10]. This study established a high degree of correlation between the results for the optimal AV interval determined by the two different methods. The mean deviation in optimal AV interval between the results from Ritter's formula and determination of stroke volume by impedance cardiography was ± 26 ms for the atrial-triggering mode, and ± 30 ms for the AV sequential mode. Kindermann et al. criticized the fact that it is possible to apply Ritter's method only for patients with ventricular stimulation.

In comparison to time-consuming and expensive RNV, and AV-interval optimization by Swan-Ganz catheterization (with the associated risks of an invasive procedure), Ritter's method offers the following advantages: it is non-invasive and can be quickly performed (approx. 5 min.). It does not require long years of echo experience, and it is cost-effective. Even with patients not readily amenable to sonographic detection, the mitral valve inflow profile is almost always qualitatively satisfactory enough to allow application of Ritter's method. The only noteworthy disadvantage of this method is the necessity for continuous ventricular stimulation: which means that it can be used only for patients with a complete AV block. Patients with only intermittent high-grade AV blocks are accordingly not suited for Ritter's method.

In our patients, the mean optimal AV interval in Group 1 (EF > 35%) was 190 ± 28.5 ms. In comparison, the optimal AV interval among the patients with chronic heart failure in Group 2 was 180 ± 35 ms. Data in the literature are not consistent on the duration of the optimal AV delay. Kindermann [10] considers AV_opt _= 88 ms ± 35 ms with atrial triggering, and AV_opt _= 143 ms ± 41 ms for the AV sequential mode. Knorre [18] has determined AV_opt _= 100.5 ± 27.8 ms for atrial triggering, and AV_opt _= 169 ± 24.5 ms for the AV sequential mode. Haskel [5] has established the best AV interval to be 150 ms. Janosik [6] considers AV_opt _= 144 ± 48 ms with atrial triggering, and AV_opt _= 176 ± 44 ms for the AV sequential mode. Ishikawa [15] has determined AV_opt _= 161 ± 26 ms.

Our results on the length of the optimal AV delay lie within the range found in the literature. The variance in data observed in some cases emphasizes the highly individual nature of the optimal AV delay: indeed, it results from the interatrial conduction period specific to each patient, and the potential delays induced by pacing versus intrinsic depolarization and conduction in a given patient [1-4].

As a result, the mean optimal AV intervals determined by us cannot be applied to other patient cohorts with the same basic disease. Individual programming of the AV interval is therefore necessary.

## Conclusion

In summary, our findings confirm that Ritter's method can be reliably applied for patients with normal and with reduced left ventricular pump function. The only prerequisite is a continuous ventricular stimulation.
